# Removal of Methylene Blue and Methyl Red from Aqueous Solutions Using Activated Carbons Obtained by Chemical Activation of Caraway Seed

**DOI:** 10.3390/molecules28176306

**Published:** 2023-08-29

**Authors:** Dorota Paluch, Aleksandra Bazan-Wozniak, Agnieszka Nosal-Wiercińska, Robert Pietrzak

**Affiliations:** 1 Department of Applied Chemistry, Faculty of Chemistry, Adam Mickiewicz University in Poznan, Uniwersytetu Poznanskiego 8, 61-614 Poznan, Poland; dorota.paluch@amu.edu.pl (D.P.); aleksandra.bazan@amu.edu.pl (A.B.-W.); robert.pietrzak@amu.edu.pl (R.P.); 2 Department of Analytical Chemistry, Institute of Chemical Sciences, Faculty of Chemistry, Maria Curie-Sklodowska University in Lublin, Maria Curie-Sklodowska Sq. 3, 20-031 Lublin, Poland

**Keywords:** activated carbon, caraway seeds, chemical activation, methylene blue, methyl red

## Abstract

In this study, activated carbons were produced through the chemical activation of caraway seeds using three different activators: Na_2_CO_3_, K_2_CO_3_, and H_3_PO_4_. A 1:2 weight ratio of precursor to activator was maintained in every instance. Comprehensive analyses were conducted on the resultant activated carbons, including elemental analysis, textural parameters determination, Boehm titration for surface oxygen functional groups, pH assessment of aqueous extracts, and quantification of ash content. The produced materials were subjected to adsorption tests for methylene blue and methyl red sodium salt from the liquid phase and the effects of adsorbent dosage, pH of the aqueous dye solution, process temperature, and adsorbent–adsorbate contact time on sorption capacity obtained. To characterize the adsorption model of the examined pollutants, both the Langmuir and Freundlich equations were employed. In addition, the sorption capacity of the obtained carbon materials against an iodine aqueous solution was assessed. The specific surface area of the obtained adsorbents ranged from 269 to 926 m^2^/g. By employing potassium carbonate to chemically activate the starting substance, the resulting activated carbons show the highest level of specific surface area development and the greatest sorption capacity against the tested impurities—296 mg/g for methylene blue and 208 mg/g for methyl red sodium salt. The adsorption rate for both dyes was determined to align with a pseudo-second-order kinetic model. The experimental adsorption data for methylene blue were well-described by the Langmuir model, whereas the Freundlich model was found to be congruent with the data pertaining to methyl red sodium salt.

## 1. Introduction

Dyes are a group of complex organic substances containing unsaturated (chromophore) bonds in their structure, which grant them the ability to absorb radiation in the visible light range (400 to 700 nm). Consequently, the color is perceived by the fraction of light that the dye does not absorb but instead reflects back [1].

Historically, color has held paramount importance in human societies. From prehistoric periods, it has served not only as a means of communication but also as a pivotal medium of artistic expression. Pigments, initially derived from mixtures of soil or other substances with water, saliva, or animal fat, were used to color both human skin and commonplace objects. Throughout history, pigments have been used in cave paintings, ceramics, and writing, and their use has increased with the development of human civilization [2]. Notably, a breakthrough came with the synthesis of the first artificial dye, Mauvein, by Henry Perkin in 1856 [3]. This innovation fueled the exponential growth of the textile industry, which, astonishingly, stands at an annual output of 8 × 10^5^ tonnes today [4]. Given its vast scale, the environmental implications of the textile industry are profound. It is responsible for generating approximately 55% of the world’s colored wastewater, amounting to nearly 200 × 10^5^ tonnes annually. This is followed by the dyeing industry at 21%, while the tanning and paper and pulp industries represent 8% and 9%, respectively [4,5]. The rampant utilization of dyes, therefore, is contributing significantly to environmental degradation, endangering ecosystems worldwide.

Presently, synthetic dyes dominate the colorant market, with estimates suggesting the commercial use of over 100,000 different synthetic dyes [6]. In the textile sector, fibers are spun into yarns, which are subsequently woven or knitted into fabrics [7]. However, during the dyeing process, up to 75% of the introduced dye is washed away by water [8]. Huge amounts of pollutants such as heavy metals, organic dyes, and surfactants enter the environment. Such contamination, especially in aquatic environments, is posing a growing threat to all living organisms and presents a significant challenge to scientists with each successive year. Moreover, a large number of the dyes used decompose slowly or not at all, which contributes to the difficulty of implementing biological wastewater treatment methods [6].

In light of these challenges, recent years have witnessed the emergence of innovative dyeing wastewater treatment methods specially designed to tackle dyes that are hard to degrade due to their intricate chemical structures. Adsorption techniques, in particular, have gained traction due to their efficacy and cost-effectiveness. Among conventional adsorbents, activated carbon is widely used due to its excellent adsorption properties [9]. In recent years, there has been increased interest in researching the production of activated carbons from agricultural and industrial wastes [10].

Activated carbon is a highly porous adsorbent lauded for its expansive surface area and potent surface reactivity. It finds applications across various industries including drinking water purification, air and gas filtration, and even the food industry [8]. In 2020, the global activated carbon market was valued at nearly $3 billion, and projections indicate a potential surge to $4.5 billion by 2028 [8]. The efficacy of activated carbon in removing contaminants from drinking water, such as organic substances, colorants, and trace chemicals, is widely recognized [10]. In 2009, Stavropoulos et al. [11] documented varying production expenses and additional economic metrics associated with the production processes of physically and chemically activated carbon. These processes were dependent on precursor materials like used tires, wood, petroleum coke, carbon black, coal, and lignite. The researchers determined production costs as follows: 2.23 USD/kg, 2.49 USD/kg, 1.08 USD/kg, 1.22 USD/kg, 1.25 USD/kg, and 2.18 USD/kg for the respective precursors. We are of the opinion that the manufacturing of activated carbons from biomass caraway seeds has the potential to lower the expenses associated with carbon adsorbent production.

Typically, commercial activated carbon is derived from coal, a depleting non-renewable resource. In response to this, there is a push to identify alternative precursors and sources. Raw materials rich in carbon, like lignocellulosic materials, lignite, polymers, and carbonaceous waste, can be utilized to produce activated carbon [12]. The use of biowaste has a positive impact on environmental protection by reducing solid waste, as well as producing low-cost activated carbons capable of removing pollutants [10]. Although many scientific papers are published each year on the preparation of carbon adsorbents from plant residues, there are currently not many papers discussing the preparation of activated carbons from caraway seeds [10].

Therefore, the aim of the study was to produce a range of activated carbons through the chemical activation of caraway seeds. Subsequently, the physicochemical attributes and sorption capabilities of the resulting carbon adsorbents towards aqueous-phase pollutants were assessed. Methylene blue and methyl red sodium salt were selected as representative organic pollutants to gauge the adsorption traits of the activated carbons.

## 2. Results and Discussion

### 2.1. Chemical Composition of the Activated Carbons

Elemental analysis was carried out for the precursor and the activated carbons obtained by chemical activation with sodium carbonate (CA_Na_), potassium carbonate (CA_K_), and 50% orthophosphoric acid(V) (CA_P_). As the data presented in Table 1 shows, the dried caraway seeds contained 52.72 wt.% carbon, 36.54 wt.% oxygen, 7.41 wt.% hydrogen, 2.89 wt.% nitrogen, and 0.44 wt.% sulfur. The weight content of ash in the caraway seeds was 4.67 wt.%. Elemental analysis of the adsorbents obtained shows that the adsorbent obtained by chemical activation of the precursor with sodium carbonate had the highest elemental carbon content (76.25 wt.%). In addition, the CA_Na_ sample also contained 4.39 wt.% nitrogen, 1.60 wt.% hydrogen, 0.55 wt.% sulfur, and 36.54 wt.% oxygen. The mineral content of this activated carbon was 2.09 wt.%. The adsorbent obtained by chemical activation of caraway seeds with orthophosphoric acid(V) had the lowest content of elemental carbon (73.76 wt.%) and nitrogen (1.71 wt.%), and the highest content of hydrogen (2.66 wt.%), sulfur (0.58 wt.%), and oxygen (21.29 wt.%). Chemical activation of the precursor with potassium carbonate leads to a sample with the lowest mineral content (0.53 wt.%). Analyzing the results shown in Table 1, it can be concluded that chemical activation of caraway seeds results in a significant increase in elemental carbon, which is due to a decrease in hydrogen and oxygen content compared to the starting material.

Ash is an impurity that can limit the adsorption capacity of activated carbon [12]. The decrease in mineral content in the CA_Na_ and CA_K_ activated carbons compared to the precursor is due to the use of washing the obtained carbon material with hydrochloric acid solution. This step was skipped in the preparation of the CA_P_ sample, therefore the % ash content in its structure is the highest at 9.79 wt.%.

**Figure 1:**
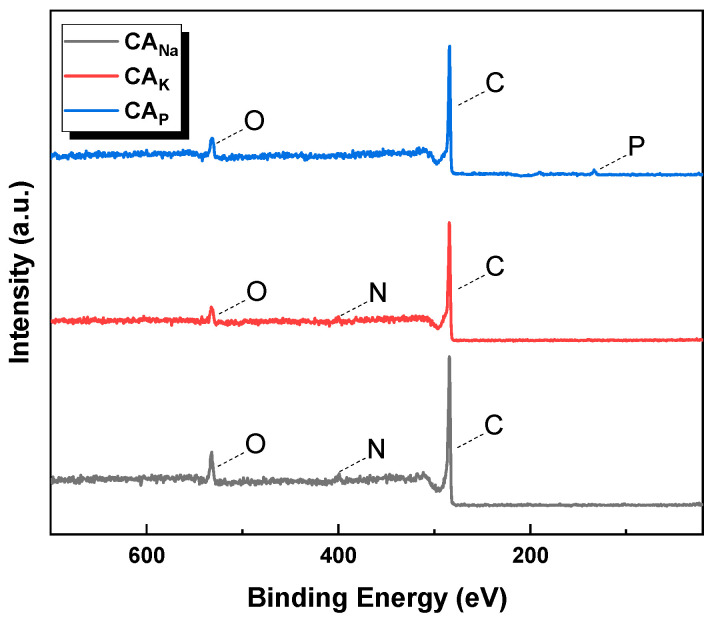
Survey XPS spectra of obtained activated carbons.

X-ray Photoelectron Spectroscopy (XPS), also known as Electron Spectroscopy for Chemical Analysis (ESCA), was used to determine the surface composition of the tested material both qualitatively and quantitatively. The spectra shown in Figure 1 confirm the results of the elemental analysis. The carbon obtained by chemical activation of the precursor with sodium carbonate exhibited the highest peak intensities originating from C1s, N1s, and O1s. This aligns with the elemental analysis results, as the CA_Na_ sample displayed the highest weight percentage of these elements. In the case of CA_P_ carbon, no peak corresponding to nitrogen was observed. However, a peak can be observed from phosphorus groupings, which are formed when the starting material is impregnated with orthophosphoric acid(V) and annealed in an inert atmosphere [13].

### 2.2. Physiochemical Properties of the Obtained Activated Carbons

Table 2 shows the textural parameters of the obtained activated carbons. The carbon obtained by chemical activation of caraway seeds with potassium carbonate had the most developed specific surface area. The surface area of this carbon measured 926 m^2^/g, and the volume of micropores was 0.65 cm^3^/g. In contrast, the average pore diameter of the CA_K_ sample was 2.80 nm. The least-developed specific surface area is that of carbon obtained by chemical activation of the starting material with sodium carbonate—269 m^2^/g. Further analysis of the data summarized in Table 2 also allows us to conclude that CA_Na_ carbon was characterized by the smallest volume of micropores and the largest average pore diameter. On the basis of the textural parameters of the tested activated carbons, it can be assumed that sodium carbonate did not allow effective development of the specific surface area. In contrast, the specific surface area of the carbon obtained by chemical activation with a 50% solution of orthophosphoric acid(V) was 46% larger than that of CA_Na_ carbon and almost 63% smaller than that of CA_K_ carbon.

Comparing the textural parameters of adsorbents obtained from caraway seeds and materials obtained in an analogous way from fennel seeds [14], it can be observed that CA_Na_ and CA_K_ samples had a less developed specific surface area compared to samples obtained from fennel. The adsorbent obtained by chemical activation of fennel seeds with potassium carbonate exhibited a specific surface area that was 124 m^2^/g higher than that of the CA_K_ sample. For activation of the precursor with potassium carbonate, the difference was 76 m^2^/g. In contrast, the CA_P_ sample obtained by activating the precursor with a 50% solution of orthophosphoric acid(V) had a specific surface area that was 62 m^2^/g higher than the material obtained in an analogous manner from fennel seeds. Thus, it can be concluded that sodium and potassium carbonates allowed the specific surface area of adsorbents obtained from fennel seeds to be developed more efficiently than those obtained from caraway seeds. However, orthophosphoric acid(V) more effectively developed the surface area of the adsorbent when caraway seeds were the precursor.

In contrast, a study by Khan et al. [13] showed that activation of custard apple fruit shell with orthophosphoric acid(V) in a weight ratio of 1:1.5 (precursor:activator) at 700 °C produced an adsorbent with a specific surface area as high as 1065 m^2^/g. This is almost twice the surface area of the CA_P_ sample. Meanwhile, activated carbon, obtained by González-García et al. [15] by activating water hyacinth stems with potassium carbonate at a weight ratio of precursor:activator of 1:2 at 550 °C, had a specific surface area of only 235 m^2^/g, which is significantly lower than the result obtained for a CA_K_ sample obtained with the same activator at an identical weight ratio but activated at 700 °C. This suggests that the use of a higher activation temperature may lead to an adsorbent with a better-developed specific surface area.

The iodine numbers of the obtained activated carbons showed correlations with the textural parameters, as shown in Table 2. The iodine adsorption number was found to be 278 mg/g for CA_Na_ carbon, 1107 mg/g for CA_K_ carbon, and 1103 mg/g for CA_P_ carbon. Such a high iodine adsorption number for carbon obtained by chemical activation of caraway seeds with a 50% solution of orthophosphoric acid(V) was most likely due to the presence of phosphorus groups on its surface, the presence of which on the surface of the CA_P_ sample is confirmed by XPS studies (Figure 1). Due to the presence of phosphorus and carboxyl functional groups on the surface of CA_P_ carbon, the efficiency of activated carbon in adsorbing inorganic pollutants such as iodine increases [16]. A similar relationship occurred with activated carbon obtained in an analogous way from fennel seeds [14].

**Figure 2:**
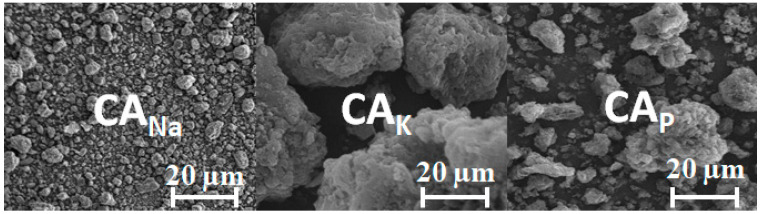
SEM images.

Figure 2 presents SEM images of the activated carbon samples, confirming the textural and morphological differences between them. Depending on the applied activator, the samples differ in pore size, shape, and number. The brighter fragments observed for activated carbons may be due to the presence of ash.

**Figure 3:**
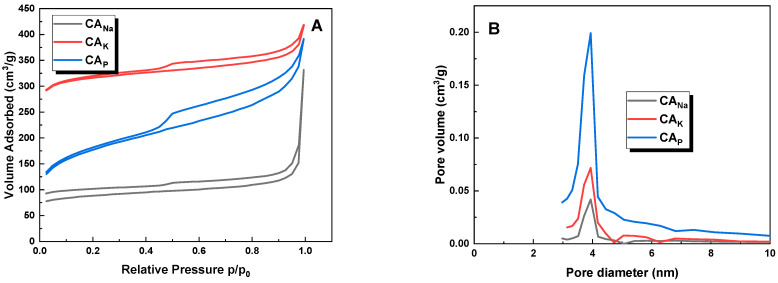
Low-temperature N_2_ adsorption-desorption isotherms (**A**) and pore size distribution (**B**) of the obtained activated carbons.

Figure 3 presents the low-temperature nitrogen adsorption/desorption isotherms (A) and pore distribution (B) for the obtained activated carbons. These isotherms can be assigned to type IV(a) according to the International Union of Pure and Applied Chemistry (IUPAC) classification [17]. Type IV(a) adsorption isotherms are typical of mesoporous materials. This type of isotherm is characterized by capillary condensation in pores with diameters in the mesoporous range (from 2 to 50 nm). The shape of the curves is confirmed by the data summarized in Table 2. The IUPAC classification defines six types of hysteresis loops, which are closely correlated with the characteristics of the pore structure and the adsorption mechanism. For all samples, the hysteresis loops shown in the graph are classified as type H4, which is typical of materials having narrow, gaping pores [17]. Type H4, according to the IUPAC classification, describes a hysteresis known as “ink-bottle” hysteresis. This is a type of hysteresis that is characteristic of materials in which narrow openings lead to much wider spaces.

The acid–base nature of the surface of activated carbons is of fundamental importance for the adsorption of contaminants from the aqueous phase due to the interactions occurring between the adsorbate and the adsorbent [16]. The acid–base characteristics of the produced activated carbons and precursor are presented in Table 3. To make these determinations, the pH of the aqueous extracts of these substances was measured, and the number of surface oxygen functional groups was determined using the Boehm method. Based on the data obtained, it can be concluded that caraway seed showed a slightly acidic pH of the aqueous extract with a value of 6.6 and a predominance of acidic (5.19 mmol/g) over basic (3.34 mmol/g) groups. Moreover, all the adsorbents obtained had a predominance of acidic over basic groupings on their surface, as confirmed by the pH values of their aqueous extracts. The most acidic character of the surface was shown by carbon obtained by activating the precursor with a 50% solution of orthophosphoric(V) acid. The pH value of this adsorbent was 3.0, while the content of acidic and basic oxygen functional groups was 2.05 and 0.35 mmol/g, respectively. The pH values for the aqueous extract were 5.3 for the CA_K_ adsorbent and 6.3 for the adsorbent activated with sodium carbonate. The surface of the CA_K_ and CA_Na_ samples contained acidic oxygen functional groups amounting to 1.60 and 2.15 mmol/g, respectively, and basic functional groups amounting to 0.35 and 0.70 mmol/g, respectively.

### 2.3. Adsorption of Dyes

Adsorption tests were carried out for the obtained activated carbons against aqueous solutions of two organic dyes: methylene blue (MB) and methyl red sodium salt (MR).

**Figure 4:**
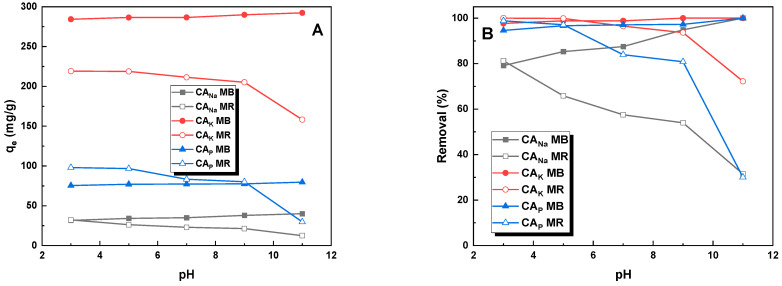
Influence of pH value on the adsorption (**A**) and efficiency of removal (**B**) of methylene blue and methyl red sodium salt (activated carbon mass: 25 mg, initial dye solution concentration for methylene blue: 20 mg/L for CA_Na_, 145 mg/L for CA_K_, and 50 mg/L for CA_P_, initial dye concentration for methyl red sodium salt: 20 mg/L for CA_Na_, 110 mg/L for CA_K_, and 50 mg/L for CA_P_, dye solution volume: 50 mL, temperature: 23 ± 1 °C).

The pH value of an aqueous solution of a dye has a significant effect on its adsorption efficiency. Due to differences in structure, organic dyes can be divided into cationic (methylene blue) and anionic (methyl red sodium salt) dyes. The presence of hydrogen and hydroxyl ions in the aqueous solution changes the charge present on the surface of the adsorbent, which can have a positive or negative effect on the sorption capacities of activated carbons. Figure 4 shows the effect of the pH of the aqueous dye solution on the sorption capacities of activated carbons obtained by the chemical activation of caraway seeds. Analyzing the data shown in the graph, it can be concluded that for the cationic dye (methylene blue), higher pH values have a positive effect on the obtained sorption capacities. However, for the sodium salt of methyl red, a definite decrease in the % removal of the dye and sorption capacities was observed with increasing pH values. An identical relationship was reported in a previous study [18]. A decrease in the sorption capacities of activated carbons against an aqueous solution of methyl red was also demonstrated in a study by Rajoria et al. [19]. This may be due to the fact that at low pH values of the solution, the surface of the adsorbent is positively charged and there are electrostatic interactions between the surface of the activated carbon and the dye molecules that enhance the phenomenon of adsorption of an anionic pollutant such as MR on the surface of activated carbons. Conversely, at higher pH values, excess hydroxyl ions can compete with MR molecules and thus block active sites present on the adsorbent surface. In the case of the second dye, the relationship observed was the opposite.

**Figure 5:**
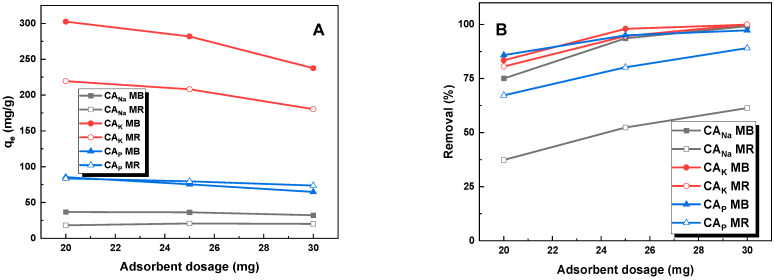
Effect of adsorbent dosage (**A**) and adsorption efficiency (**B**) on adsorption of methyl red sodium salt and methylene blue (volume of dye solution: 50 mL, initial dye solution concentration for methylene blue: 20 mg/L for CA_Na_, 145 mg/L for CA_K_, and 50 mg/L for CA_P_, initial dye concentration for methyl red sodium salt: 20 mg/L for CA_Na_, 110 mg/L for CA_K_, and 50 mg/L for CA_P_, temperature: 23 ± 1 °C).

Figure 5 shows the effect of the dosage of activated carbon on its sorption capacities against the tested contaminants. The data presented in Figure 5B demonstrate that for each adsorbent, the maximum percentage of pollutant removal occurred when the mass of activated carbon was 30 mg. This fact can be explained by the greater availability of active sites due to the increased surface area of the adsorbent. Increasing the mass of the adsorbent at a constant dye concentration provides more available adsorption sites for the adsorbate and thus increases the removal rate [20]. Nonetheless, there was a reduction in the dye quantity per unit mass of adsorbent, leading to a decrease in sorption capacity as depicted in Figure 5A. Therefore, in order to optimize the results, an adsorbent mass of 25 mg was assumed for further studies.

**Figure 6:**
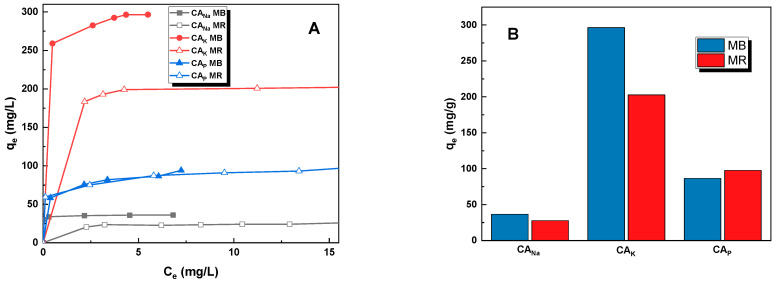
Effect of initial concentration (**A**) (activation carbon mass: 25 mg, dye concentration: for methylene blue: 15–30 mg/L for CA_Na_, 120–155 mg/L for CA_K_, and 20–55 mg/L for CA_P_, dye concentration for methyl red sodium salt: 10–40 mg/L for CA_Na_, 90–120 mg/L for CA_K_, and 20–65 mg/L for CA_P_, dye solution volume: 50 mL, temperature: 23 ± 1 °C) and experimental maximal sorption capacities (**B**) of the obtained activated carbons against methylene blue and methyl red sodium salt.

Figure 6 shows the adsorption isotherms and sorption capacities for the obtained samples against the tested dyes. The presented data indicate that the CA_K_ carbon obtained by chemically activating caraway seeds with potassium carbonate demonstrated the best sorption abilities against the tested pollutants. The sorption capacities of this sample against an aqueous solution of methylene blue and methyl red sodium salt were 296 mg/g and 203 mg/g, respectively.

These values far exceed the results obtained for the other two adsorbents. The sorption capacity of the CA_Na_ sample was the lowest of all the obtained carbon adsorbents—37 and 28 mg/g for MB and MR, respectively. The CA_P_ carbon obtained by chemical activation of the precursor with orthophosphoric acid(V) had better adsorption capacity for methyl red sodium salt than for methylene blue (q_e_ = 98 mg/g for MR, q_e_ = 87 mg/g for MB). This is due to the fact that orthophosphoric acid(V) leads to an adsorbent with a distinctly acidic pH of the aqueous extract (Table 3), resulting in a strongly acidic system that favors the adsorption of methyl red sodium salt [16].

Comparing the method of obtaining and sorption capacities of the obtained materials against MB and MR using various adsorbents described in the literature (Table 4), it can be concluded that chemical activation of fennel seeds [14] leads to materials with higher sorption capacities against methylene blue than chemical activation of caraway seeds under identical conditions. In the case of the activation of caraway seeds with orthophosphoric acid(V), an adsorbent with a better-developed specific surface area was obtained than in the case of the activation of fennel seeds under identical conditions. However, the material obtained from caraway seeds, despite a better-developed specific surface area, has been found to have a much lower sorption capacity. Comparing CA_P_ carbon with the adsorbent obtained by chemical activation of corn stigmata [21] using the same activator, precursor-to-activator ratio, and temperature, it can be observed that the adsorbent obtained from corn stigmata has a specific surface area that is 240 m^2^/g higher. Moreover, the sorption capacity of this adsorbent against methylene blue is almost 3.5-fold higher than that of the CA_P_ sample. In contrast, activated carbon obtained from custard apple fruit shell by chemical activation with orthophosphoric acid(V) at 700 °C [13] shows a sorption capacity against methyl red of 435 mg/g, which is almost 4.5 times higher than the sorption capacity of CA_P_ carbon. Such a large difference between the sorption capacities is due to the better-developed specific surface area of the adsorbent obtained from the custard apple fruit shell, which is probably the result of using a higher activation temperature.

Comparing the carbon obtained by the chemical activation of caraway seeds with potassium carbonate with the adsorbent obtained by the chemical activation of pineapple peel with KOH [22] at the same temperature, it can be observed that despite the better developed specific surface area of 1160 m^2^/g, it shows weaker sorption capacities against MB and MR, which are 165 and 95 mg/g, respectively. Chemical activation of waste after supercritical extraction of green tea leaves with sodium carbonate [17] leads to adsorbents with sorption capacities towards MR and MB that are 50 and 48 mg/g higher, respectively, than those of CA_Na_ carbon. It is worth noting that the adsorbent obtained from green tea leaves was obtained under identical conditions to those of the CA_Na_ sample and has a specific surface area that is 15 m^2^/g less.

**Figure 7:**
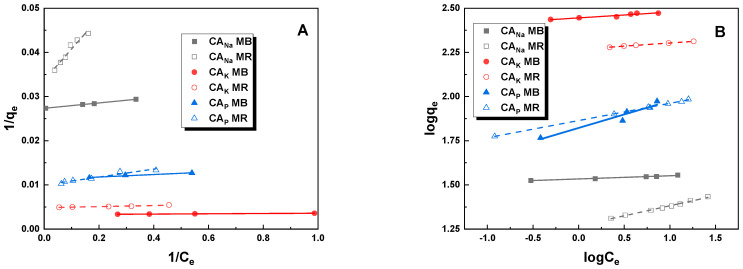
Linear fitting for methylene blue and methyl red sodium salt on obtained activated carbons to Langmuir (**A**) and Freundlich (**B**) models.

The experimentally obtained isotherms for methylene blue and methyl red sodium salt were used to calculate the characteristic parameters for two models—Langmuir and Freundlich. The fitting graphs obtained for the linear Langmuir and Freundlich models are shown in Figure 7. Comparison of the experimental data with the predictions of a particular model provides information on the mechanism of adsorption and interactions between the adsorbent and adsorbate. Based on the values shown in Table 4, it can be seen that a better fit to the Langmuir model (R^2^ = 0.997–0.999) than to the Freundlich model (0.973–0.898) is observed for methylene blue. Therefore, adsorption of an aqueous solution of methylene blue on the obtained activated carbons followed the Langmuir isotherm, and thus an adsorption monolayer was formed on the surface of the adsorbents. However, when an aqueous solution of methyl red sodium salt was adsorbed onto the tested samples, a completely different relationship was observed. Therefore, it can be concluded that the adsorption process occurs with the formation of an adsorption multilayer [23]. Further analysis of the data summarized in Table 5 allows us to conclude that in the case of MB adsorption, the calculated values of q_max_ were very close to the values obtained experimentally. The Langmuir constant (K_L_) was highest for CA_Na_ carbon on which methylene blue adsorption occurs (13.24 L/mg). This means that the strongest interactions occur between the MB dye molecule and CA_Na_ carbon during the adsorption process. In contrast, for the adsorption of methyl red sodium salt on the same adsorbent, the Langmuir constant was only 0.48 L/mg.

The weakest adsorbent–adsorbate bonds occur for CA_P_-MR, with a K_L_ constant of only 0.28 L/mg. It can also be noted that for each of the adsorbents, the Langmuir constant for methylene blue was higher than that for methyl red sodium salt. The R_L_ for each of the tested materials ranges from 0 to 1, which means that the adsorption of the tested pollutants on activated carbons is favorable [23]. The K_F_ parameter present in the Freundlich isotherm equation determines the selectivity of the adsorption process [24]. According to the data presented in Table 5, it can be inferred that the CA_K_ sample exhibited the highest selectivity for the aqueous solution of the organic dye. The K_F_ constant values for MB and MR were 272.79 and 176.81 mg/g(L/mg)^1/n^, respectively. In contrast, the lowest values of this parameter were recorded for the CA_Na_ sample. It is worth noting that the K_F_ value correlates with the experimental sorption capacity, and in the case of CA_Na_ and CA_K_ carbons, it was higher for methylene blue, while in the case of CA_P_ carbon, it was higher for methyl red sodium salt. The highest 1/n coefficient was recorded for MR adsorption on CA_Na_ carbon, indicating the greatest heterogeneity of the system. The values of the constant for the other systems were in the range of 0.01–0.05.

**Figure 8:**
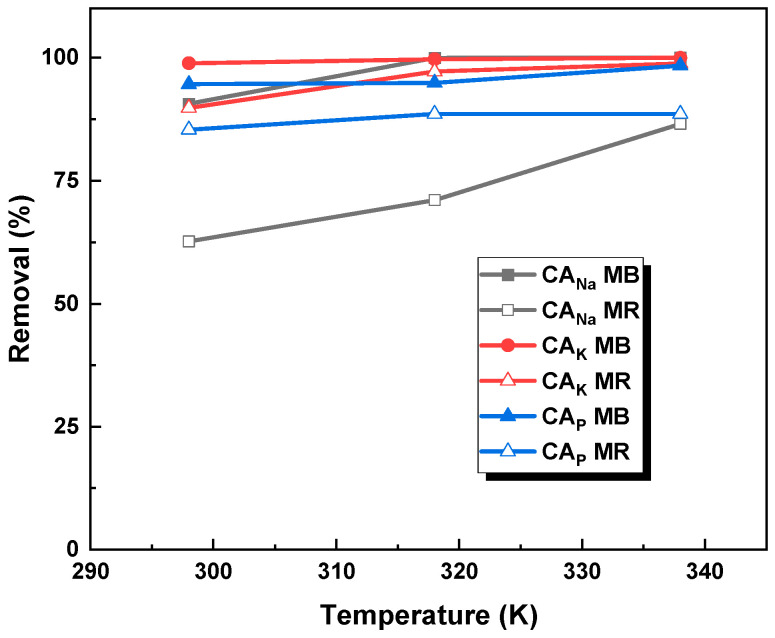
Influence of temperature on the removal of methylene blue and methyl red sodium salt (activated carbon mass: 25 mg, initial dye solution concentration for methylene blue: 20 mg/L for CA_Na_, 145 mg/L for CA_K_, and 50 mg/L for CA_P_, initial dye concentration for methyl red sodium salt: 20 mg/L for CA_Na_, 110 mg/L for CA_K_, and 50 mg/L for CA_P_, dye solution volume: 50 mL).

The next phase of the research involved assessing how temperature influenced the effectiveness of removing methylene blue and methyl red sodium salt. To determine the nature of the adsorption process, three temperature variants were used: 298 K, 318 K, and 338 K. From the data shown in Figure 8, it can be concluded that the temperature has little effect on the adsorption of MB/MR on the obtained activated carbons, which is beneficial from an economic point of view.

Within the temperature range of 298 K to 338 K, a rise in the % removal of the tested dyes was noted, ranging from 1.2 to 10 percentage points. Notably, in the context of the adsorption of methyl red sodium salt on CA_Na_ carbon, an exceptional increase in dye removal by 24 percentage points occurred specifically between 298 K and 338 K.

The data obtained experimentally were used to calculate the thermodynamic parameters (Table 6). Based on the compiled results, it can be concluded that the adsorption of the dye on all the obtained adsorbents is spontaneous, as confirmed by the values of ∆G^0^ and K_d_ [25]. Considering the positive enthalpy values of ∆H^0^, it can be inferred that the reactions occurring between the organic dye and the adsorbent are endothermic. Moreover, the adsorbent/methylene blue systems show higher values of ∆H^0^, indicating that these systems have higher internal energy than the analogous systems containing methyl red sodium salt. Based on the entropy value of ∆S^0^, it can be determined that systems containing an aqueous solution of methylene blue generally exhibit higher disorder than analogous systems containing an aqueous solution of methyl red sodium salt. However, the exception is CA_K_ carbon, where the opposite relationship is observed.

**Figure 9:**
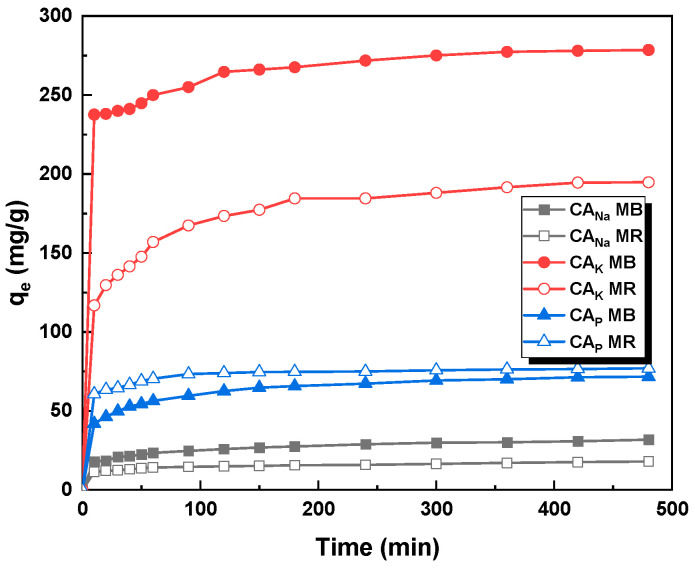
Influence of adsorbent–adsorbate contact time on the sorption capacities of the activated carbons obtained (activated carbon mass: 25 mg, initial dye solution concentration for methylene blue: 20 mg/L for CA_Na_, 145 mg/L for CA_K_, and 50 mg/L for CA_P_, initial dye concentration for methyl red sodium salt: 20 mg/L for CA_Na_, 110 mg/L for CA_K_, and 50 mg/L for CA_P_, dye solution volume: 50 mL, temperature: 23 ± 1 °C).

The study also investigated the effect of the contact time between the adsorbent and the adsorbate on the efficiency of organic dye removal by the obtained activated carbons. Based on the data shown in Figure 9, it can be concluded that the adsorption equilibrium is established after about 120–150 min from the start of the process. This is advantageous from an economic point of view. The obtained results were used to determine the mechanism of adsorption of dyes on the obtained samples (Table 7).

**Figure 10:**
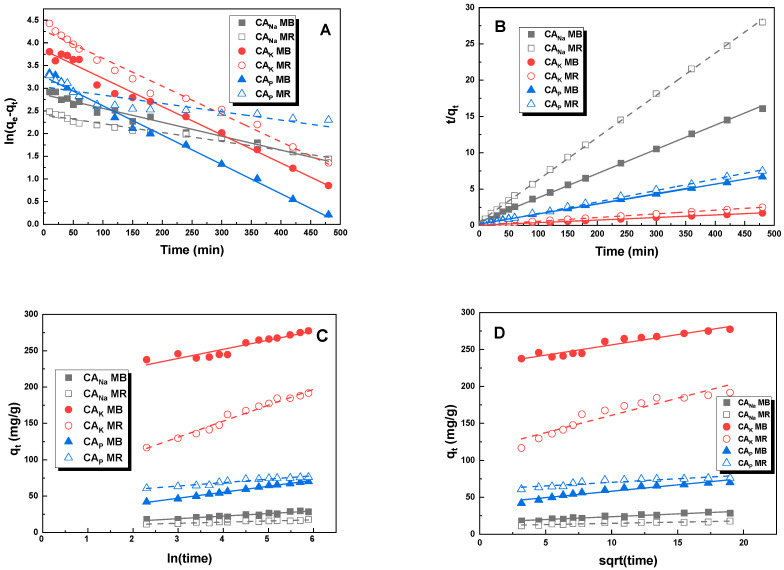
Linear fitting for methylene blue and methyl red sodium salt on obtained activated carbons to pseudo-first-order (**A**), pseudo-second-order (**B**), Elovich (**C**), and intraparticle diffusion model (**D**).

The resulting graphs for the pseudo-first-order, pseudo-second-order, Elovich, and intraparticle diffusion linear kinetic models are shown in Figure 10. The constants for the four kinetic models—pseudo-first-order, pseudo-second-order (Table 7), Elovich, and intraparticle diffusion (Table 8)—were determined. The correlation coefficient R^2^ for the pseudo-first-order model is in the range of 0.770–0.986, for Elovich 0.886–0.995, for intraparticle diffusion 0.830–0.968, while for the pseudo-second-order model, the value is not less than 0.997.

Based on this, it can be concluded that for all activated carbons, the adsorption of each dye occurs according to the pseudo-second-order model. This conclusion is further supported by the theoretically calculated values of sorption capacities, which, in the case of the pseudo-second-order model are closer to the experimental results. This suggests that for MR and MB adsorption on the activated carbons obtained, the rate of filling the available active sites by adsorbate is proportional to the square of the number of unfilled sites [26].

### 2.4. Mechanism of Adsorption

**Figure 11:**
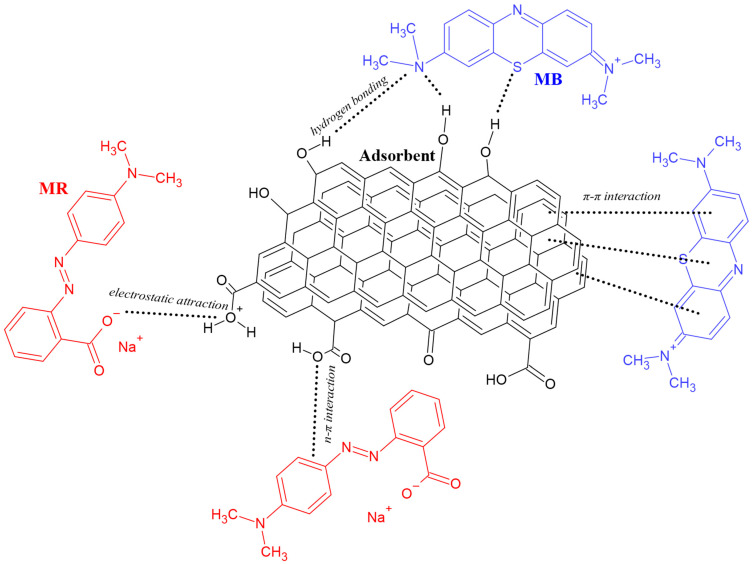
Examples of mechanisms of methylene blue and methyl red sodium salt uptake by adsorption on the surface of activated carbon.

To elucidate the mechanism of adsorption of methylene blue and methyl red sodium molecules on the obtained activated carbons, it is first necessary to determine the acidity dissociation constant (pK_a_) for each dye. These values are 3.14 and 4.80 for MB and MR, respectively [27,28]. This is crucial because the pH of the solution plays a key role in the electrostatic interaction between the adsorbent surface and the adsorbed dye molecule. The net charge on the adsorbent surface is determined by the isoelectric point and has a key effect on the nature of the interaction [29]. The dye can be adsorbed in one of the following ways: (i) interactions with either the positively or negatively charged surface of the adsorbent via electrostatic attraction, (ii) π-π interactions between aromatic rings present in the structure of both the adsorbate and the adsorbent, (iii) hydrogen bonds, or (iv) interactions between an aromatic ring and a heteroatom containing free electron pairs (Figure 11) [29].

## 3. Materials and Methods

### 3.1. Materials

Caraway seeds were used as a precursor to obtain activated carbons. The volatile content of the material used was 6.69 wt.%, moisture was 5.58 wt.%, and ash was 4.67 wt.%. The precursor was subjected to 24 h of drying. The starting material thus prepared was subjected to chemical activation using three activating agents: sodium carbonate, potassium carbonate, and a 50% solution of orthophosphoric acid(V). Methylene blue (MB) and methyl red sodium salt (MR) were used as examples of organic impurities (Table 9) and iodine as an example of an inorganic impurity. The analytical grade methylene blue (MB) and methyl red sodium salt (MR) were obtained from Merck (Darmstadt, Germany). The rest of the chemicals employed were acquired from Sigma-Aldrich (Burlington, MA, USA) at analytical grades.

### 3.2. Characterization Techniques

Textural properties of the prepared activated carbons were determined from nitrogen adsorption/desorption isotherms measured at 77 K using an AutosorbiQ analyzer from Quantachrome Instruments (Boynton Beach, FL, USA). Prior to adsorption measurements, the carbons were degassed under vacuum for 12 h at 300 °C. Specific surface area was calculated from nitrogen adsorption isotherm data using the Brunauer, Emmett, and Teller method. The total pore volume (V_t_) was estimated from the volume of nitrogen adsorbed at a relative pressure of p/p^0^ = 0.99, which corresponds to the equilibrium pressure divided by the saturation pressure, and converted to the volume of liquid nitrogen at a specific temperature. The average pore size (D) was calculated from the formula: D = 4V_t_/S_BET_, where S_BET_ is the specific surface area of carbon. The pores were assumed to be cylindrical in shape. Standard ash analysis was performed in accordance with ASTM D2866-94 (2004). A Thermo Scientific FLASH 2000 Elemental Analyzer (OEA, Thermo Fisher Scientific, Waltham, MA, USA) was used to determine the elemental composition of the precursor and activated carbons.

X-ray photoelectron spectroscopy (XPS) was carried out using an ultra-high-vacuum photoelectron spectrometer based on a Phoibos150 NAP analyzer (Specs, Berlin, Germany). The analytical chamber was operated under a vacuum at a pressure close to 5 × 10^−9^ mbar, and the sample was irradiated with monochromatic Al Kα radiation (1486.6 eV). Any charge that occurred during the measurements (due to incomplete neutralization of ejected surface electrons) was compensated for by rigidly shifting the entire spectrum by the distance needed to set the C1s binding energy assigned to the random carbon to an assumed value of 284.8 eV.

SEM images were obtained using a scanning electron microscope (PHILIPS, Eindhoven, The Netherlands) in the following conditions: working distance of 14 mm, acceleration voltage of 15 kV, and digital image recording by DISS.

For the precursor and activated carbons, the content of surface acidic and basic functional groups was determined using the Boehm method. The pH of the aqueous extracts for all samples was measured using an Elmetron pH meter, model CP-401 (ELMETRON, Zabrze, Poland). In addition, the iodine adsorption number was determined for the obtained carbon adsorbents [30].

### 3.3. Preparation of Activated Carbons

The dried caraway seeds were divided into three parts, each of which was impregnated with one of three activators: Na_2_CO_3_ (CA_Na_), K_2_CO_3_ (CA_K_), or a 50% solution of H_3_PO_4_ (CA_P_). Regardless of the activator used, the ratio of precursor to activator was 1:2. The impregnated samples were then placed in a tube oven and heated from room temperature to 500 °C for CA_P_ or to 700 °C for CA_Na_ and CA_K_. Once the desired temperature was reached, the samples were thermostated for 45 min. The chemical activation process was carried out under a nitrogen atmosphere with a flow rate of 330 mL/min. After the process, the samples were cooled to room temperature. The CA_Na_ and CA_K_ samples were both pre-cleaned with a hot 5% hydrochloric acid solution and then neutralized with approximately 10 liters of boiling distilled water until a neutral pH filtrate was obtained. The CA_P_ sample was rinsed with boiling distilled water. The activated carbons thus obtained were then dried to a constant weight before being sieved through a 0.09 mm sieve.

### 3.4. Adsorption Studies

Stock aqueous solutions of methylene blue and methyl red sodium salt (MB and MR 1000 mg/L) were prepared, from which solutions with concentrations ranging from 10–200 mg/L were then prepared. For the adsorption process, 25 mg of each type of carbon was mixed with 50 mL of a dye solution of the specified concentration. The samples were vigorously mixed for 24 h on a shaker (Heidolph, Schwabach, Germany) at 300 rpm. After this time, samples were collected with a syringe and centrifuged for 10 min in a laboratory centrifuge (OHAUS, Parsippany, NJ, USA). The concentration of methylene blue and methyl red sodium salt in solution was determined spectrophotometrically at a maximum wavelength λ_max_ of 665 nm for methylene blue and 430 nm for methyl red sodium salt, using a Carry 100 Bio spectrophotometer (Agilent, Santa Clara, CA, USA). The amount (*q_e_*) of dye adsorbed on the adsorbents tested was calculated using the formula:

(1) $$q_{e}=\frac{C_{0}-C_{e}}{m}\times V$$

where *C*_0_—initial concentration of dye solution (mg/L); *C_e_*—concentration of dye remaining in solution in equilibrium (mg/L); *m*—weight of sample (g); and *V*—volume of dye solution (L).

The effect of activated carbon mass (20–30 mg) on its sorption capacities towards the dyes tested was investigated. Each sample was flooded with 50 mL of aqueous MB or MR solution. The concentrations of methylene blue were 20 mg/L for sample CA_Na_, 40 mg/L for sample CA_P_, and 145 mg/L for sample CA_K_. In contrast, for methyl red sodium, the concentrations for the CA_Na_, CA_P_, and CA_K_ samples were 20, 50, and 110 mg/L, respectively. The effect of the pH value (pH values 3–11) of the aqueous methylene blue solution and methyl red sodium salt on the sorption capacity of the obtained activated carbons was determined (BlueLine 25 pH electrode (SI Analytics, Weilheim, Germany). Measurements were carried out for 25 mg samples. Each was flooded with 50 mL of aqueous MB or MR solution at concentrations analogous to those used to study the effect of adsorbent dose. The effect of process temperature on the adsorption of methylene blue by the obtained carbon materials was also investigated. A 25 mg sample was weighed and flooded with dye solutions at concentrations analogous to those used to study the effect of the dose and pH value of the aqueous dye solution. MB sorption tests on the obtained activated carbons were carried out at three different temperatures: 298 K, 318 K, and 338 K. For the studies of the effect of adsorbent dose, pH of the dye solution, and process temperature, the samples were shaken for 24 h on a shaker (Heidolph, Schwabach, Germany) at a rate of 300 rpm/min. Subsequently, spectrophotometric measurements were performed.

In order to determine the adsorption mechanism of methylene blue and the sodium salt of methyl red on the obtained activated carbons, two models have been used: Langmuir (2) and Freundlich (3). The Langmuir isotherm postulates that the surface of an adsorbent has a limited number of adsorption sites, known as active centers, each of which can adsorb a single adsorbate molecule, forming an adsorption monolayer. It is assumed that there are no intermolecular interactions within the adsorbate [31]. In terms of the Freundlich isotherm, the assumption involves the formation of a multilayer adsorption film on the surface of the adsorbent. This model is commonly used to describe heterogeneous systems [32].

(2) $$\frac{C_{e}}{q_{e}}=\frac{1}{K_{L}\times q_{max}}+\frac{C_{e}}{q_{max}}$$

where *C_e_*—concentration of dye remaining in solution in equilibrium (mg/L), *q_e_*—amount of dye adsorbed (mg/g), *K_L_*—Langmuir constant (L/mg), and *q_max_*—adsorption capacity of the monolayer (mg/g).

(3) $$logq_{e}=logK_{F}+\frac{1}{n}\times logC_{e}$$

where *q_e_*—amount of dye adsorbed (mg/g), *K_F_*—Freundlich constant (mg/g(L/mg)^1/n^), 1/*n*—constant related to the affinity of the adsorbate for the adsorbent, and *C_e_*—concentration of dye remaining in solution in equilibrium (mg/L).

Langmuir’s isotherm model can be characterized using a dimensionless separation factor *R_L_*:

(4) $$R_{L}=\frac{1}{1+C_{0}{\times K}_{L}}$$

The value of *R_L_* provides information as to whether the adsorption is unfavorable (*R_L_* > 1), favorable (0 < *R_L_* < 1), linear favorable (*R_L_* = 1), or irreversible (*R_L_* = 0) [33].

### 3.5. Thermodynamic Study

Sorption tests were also conducted using methylene blue and methyl red sodium salt on the obtained activated carbons at different temperatures—298 K, 318 K, and 338 K. Thermodynamic parameters such as Gibbs free energy, enthalpy, entropy, and thermodynamic equilibrium constant were determined using the following formulas for the calculations:

(5) $$∆G^{0}=-RTlnK_{d}$$

(6) $$∆G^{0}=∆H^{0}-T∆S^{0}$$

(7) $$lnK_{d}=\frac{∆S^{0}}{R}-\frac{∆H^{0}}{RT}$$

where Δ*G*^0^—Gibbs free energy, *R*—universal constant (8.314 J/mol × K), *T*—temperature (K), Δ*H*^0^—enthalpy change, Δ*S*^0^—entropy change, *K_d_*—is the dimensionless thermodynamic adsorption constant, *C_e_*—concentration of dye remaining in solution in equilibrium (mg/L), and *q_e_*—amount of dye adsorbed (mg/g) [34].

### 3.6. Adsorption Kinetics

In order to determine the kinetics of adsorption of an aqueous solution of methylene blue and methyl red sodium salt on the produced activated carbons, a pseudo-first-order kinetic model, proposed by Lagergren (8), a pseudo-second-order model, developed by Ho–McKay (9), an Elovich (10), and an Intraparticle diffusion (IPD) (11) were used. The Lagergren model is formulated to describe the adsorption rate, which is directly proportional to the difference between the equilibrium and instantaneous concentration of the adsorbate adsorbed on the adsorbent [26]. Conversely, the Ho–McKay model, as assumed, supposes that the rate at which available active sites are filled by the adsorbate is proportional to the square of the number of unfilled sites [26]. The Elovich model primarily focuses on the initial adsorption rate and can provide information about the surface adsorption process and active site availability [35]. However, the intraparticle diffusion model provides insights into the rate of mass transfer within the porous adsorbent particles [36].

(8) $$\log(q_{e}-q_{t})=logq_{e}-\frac{k_{1}}{2.303}t$$

(9) $$\frac{t}{q_{t}}=\frac{1}{k_{2}q_{e^{2}}}+\frac{t}{q_{e}}$$

(10) $$q_{t}=\frac{1}{\beta }ln(1+\alpha \beta t)$$

(11) $$q_{t}=k_{id}t^{1/2}+C$$

where *q_e_*—amount of dye adsorbed at equilibrium (mg/g), *q_t_*—amount of adsorbate absorbed in a particular time (mg/g), *t*—time (min), *k*_1_—pseudo-first-order adsorption constant (1/min), *k*_2_—pseudo-second-order adsorption constant (g/mg × min). *α* is the Elovich initial sorption rate constant (mg/g × min), *β* is the Elovich desorption constant (g/mg); *k_id_* is the intraparticle diffusion constant (mg/g × min^1/2^), and *C* is the IPD model’s boundary layer constant (mg/g) [37].

The experimental uptake capacities were compared to those calculated from the models by using the Chi-square test:

(12) $$Reduced\;Chi-square=\sum \frac{{\left(q_{e}-q_{e,m}\right)}^{2}}{q_{e,m}}\;$$

where *q_e_* is the experimental uptake capacity at equilibrium (mg/g) and *q*_*e*__,_*_m_* is the modeled uptake capacity at equilibrium (mg/g).

## 4. Conclusions

The presented study showed that caraway seeds can be used as a precursor of effective activated carbons obtained by chemical activation. The adsorbents obtained show different physicochemical and sorption properties depending on the activator used. Elemental analysis of the carbonaceous materials showed that the chemical activation of caraway seeds leads to an increase in elemental carbon content. The specific surface area of the obtained activated carbons ranges from 269 to 926 m^2^/g and the iodine numbers for the samples obtained range from 278 to 1107 mg/g. Dose effect studies have established that with increasing sample mass, the sorption capacity of the adsorbent decreases, while the % removal of the contaminant from the aqueous solution increases. This is due to the fact that with a higher mass of carbon, the number of active sites increases, allowing more dye molecules to be adsorbed. The adsorption of methylene blue on activated carbons obtained by chemical activation of caraway seeds follows the Langmuir model, while the adsorption of methyl red sodium follows the Freundlich model. It was found that as the pH value of the aqueous methylene blue solution increases, the sorption capacity of the activated carbons tested increases. In contrast, an inverse relationship was observed for an aqueous solution of methyl red sodium salt. The calculated values of the thermodynamic parameters established that the adsorption of the dyes on the obtained adsorbents is endothermic and spontaneous. It was also established that higher entropy values usually occur in systems containing methylene blue solution. On the basis of kinetic studies, it was determined that the adsorption of both dyes on the tested activated carbons follows a pseudo-second-order model. It was shown that chemical activation of the precursor with potassium carbonate leads to an adsorbent with the most favorable textural parameters and the highest sorption capacity towards the pollutants tested.

## Figures and Tables

**Table 1:** Elemental composition and content of ash of the obtained adsorbents (wt.%).

Sample	% N	% C	% H	% S	% O *	% Ash
Caraway seed	2.89	52.72	7.41	0.44	36.54	4.67
CA_Na_	4.39	76.25	1.60	0.55	17.21	2.09
CA_K_	2.83	74.89	1.91	0.23	20.14	0.53
CA_P_	1.71	73.76	2.66	0.58	21.29	9.79

*—By difference; method error ≤0.3%.

**Table 2:** Textural parameters and iodine number of the obtained activated carbons.

Sample	Surface Area ^1^ (m^2^/g)		Pore Volume (cm^3^/g)		V_m_/V_t_	Average Pore Size (nm)	Iodine Number (mg/g)
	Total	Microporous	Total	Microporous			
CA_Na_	269	224	0.55	0.12	0.22	7.61	278
CA_K_	926	865	0.65	0.47	0.73	2.80	1107
CA_P_	580	411	0.67	0.23	0.34	4.15	1103

^1^ Error range between 2–5%.

**Table 3:** Acid–base properties of the obtained activated carbons.

Sample	Acidic Oxygen Functional Groups (mmol/g)	Basic Oxygen Functional Groups (mmol/g)	pH
Caraway seed	5.19	3.34	6.6
CA_Na_	2.15	0.70	6.3
CA_K_	1.60	0.35	5.3
CA_P_	2.05	0.35	3.0

**Table 4:** Comparison of the activated carbons obtained from various reported adsorbents.

Precursor	Activator	Precursor: Activator Ratio	Activation Temperature (°C)	Total Surface Area (m^2^/g)	Dye	Sorption Capacity (mg/g)	Source
Caraway seed	Na_2_CO_3_	1:2	700	269	MR	28	This study
					MB	37	
	K_2_CO_3_	1:2	700	926	MR	203	
					MB	296	
	H_3_PO_4_	1:2	500	580	MR	98	
					MB	87	
Fennel seed	Na_2_CO_3_	1:2	700	345	MB	77	[14]
	K_2_CO_3_	1:2	700	1052	MB	474	
	H_3_PO_4_	1:2	500	518	MB	122	
Pineapple peel	KOH	1:3	700	1160	MR	95	[12]
					MB	165	
Green tea leaves	Na_2_CO_3_	1:2	700	254	MR	70	[18]
					MB	85	
Corn Stigmata	H_3_PO_4_	1:2	500	820	MB	331	[21]
Custard apple fruit shell	H_3_PO_4_	1:1.5	700	1065	MR	435	[13]

**Table 5:** Values of constants determined for linear Langmuir and Freundlich models for methylene blue and methyl red sodium salt.

Sample	Dye	q_e_ (mg/g)	Langmuir					Freundlich			
			R^2^	q_max_ (mg/g)	K_L_ (L/mg)	Reduced Chi- Square	R_L_	R^2^	K_F_ (mg/g(L/mg)^1/n^)	1/n	Reduced Chi- Square
CA_Na_	MB	36	0.999	36	13.24	6.87 × 10^−10^	6.02 × 10^−3^	0.973	34.72	0.02	4.66 × 10^−6^
CA_K_		296	0.997	298	1.36	5.96 × 10^−11^	4.80 × 10^−3^	0.898	272.79	0.05	2.53 × 10^−5^
CA_P_		94	0.997	94	1.91	3.28 × 10^−8^	1.04 × 10^−2^	0.967	69.18	0.01	5.34 × 10^−4^
CA_Na_	MR	28	0.948	30	0.48	6.60 × 10^−7^	4.91 × 10^−2^	0.993	18.97	0.10	1.43 × 10^−5^
CA_K_		202	0.907	208	3.72	3.14 × 10^−9^	1.32 × 10^−3^	0.998	176.81	0.05	8.44 × 10^−7^
CA_P_		87	0.935	120	0.28	1.28 × 10^−7^	5.25 × 10^−2^	0.999	73.40	0.10	5.95 × 10^−6^

**Table 6:** Thermodynamic parameters of adsorption of aqueous solution of methylene blue and methyl red sodium salt on the obtained carbon adsorbents.

Sample	Dye	Temperature (K)	K_d_	∆G^0^ (kJ/mol)	∆H^0^ (kJ/mol)	∆S^0^ (J/mol × K)
CA_Na_	MB	298	20.85	−7.34	217.16	753.96
		318	5.17 × 10^3^	−23.04		
		338	6.67 × 10^5^	−37.44		
CA_K_		298	177.36	−12.83	28.06	137.20
		318	361.54	−17.22		
		338	677.42	−15.93		
CA_P_		298	29.05	−8.82	25.54	113.70
		318	55.55	−9.55		
		338	98.37	−13.50		
CA_Na_	MR	298	3.00	−2.98	27.93	102.88
		318	6.10	−4.20		
		338	11.40	−7.17		
CA_K_		298	18.61	−7.11	47.63	184.15
		318	62.36	−11.23		
		338	181.09	−14.44		
CA_P_		298	12.10	−6.07	6.12	41.26
		318	14.13	−7.24		
		338	16.20	−7.69		

**Table 7:** Kinetic model parameters for methylene blue and methyl red sodium salt.

Sample	Dye	q_e_ (mg/g)	Pseudo-First-Order Kinetic				Pseudo-Second-Order Kinetic			
			k_1_ (1/min)	R^2^	q_e/cal_ (mg/g)	Reduced Chi- Square	k_2_ (g/mg × min)	R^2^	q_e/cal_ (mg/g)	Reduced Chi- Square
CA_Na_	MB	37	5.94 × 10^−6^	0.956	17	6.19 × 10^−3^	1.90 × 10^−3^	0.998	33	4.02 × 10^−2^
CA_K_		277	1.74 × 10^−5^	0.970	47	1.42 × 10^−2^	7.32 × 10^−3^	0.999	279	1.47 × 10^−4^
CA_P_		73	1.33 × 10^−5^	0.986	26	9.76 × 10^−3^	2.54 × 10^−3^	0.999	73	6.62 × 10^−3^
CA_Na_	MR	23	3.83 × 10^−6^	0.938	11	2.87 × 10^−3^	3.32 × 10^−3^	0.997	18	4.70 × 10^−2^
CA_K_		201	2.38 × 10^−3^	0.949	67	1.77 × 10^−2^	2.93 × 10^−3^	0.999	199	6.23 × 10^−4^
CA_P_		76	7.86 × 10^−6^	0.770	23	2.76 × 10^−2^	9.01 × 10^−3^	0.999	74	9.30 × 10^−3^

**Table 8:** Elovivch and intraparticle diffusion model parameters for methylene blue and methyl red sodium salt.

Sample	Dye	Elovich				Intraparticle Diffusion Model			
		*α* (mg/g × min)	R^2^	*β* (g/mg)	Reduced Chi- Square	k_id_ (mg/g × min^1/2^)	R^2^	C (mg/g)	Reduced Chi- Square
CA_Na_	MB	0.267	0.948	28.72	1.13	0.763	0.927	16.12	18.81
CA_K_		0.080	0.886	351.28	26.62	2.78	0.920	228.49	18.81
CA_P_		0.124	0.995	143.16	0.48	1.73	0.930	40.99	1.28
CA_Na_	MR	0.623	0.927	207.50	0.29	4.63	0.897	114.50	68.62
CA_K_		0.046	0.978	431.06	15.11	0.33	0.847	11.44	0.56
CA_P_		0.202	0.941	11.49	1.80	0.968	0.830	60.65	5.34

**Table 9:** Characteristics of studied dyes.

Dye	Chemical Formula	Structure	Mass (g/mol)	λ_max_ (nm)
Methylene blue	[C_16_H_18_N_3_S]^+^Cl^−^	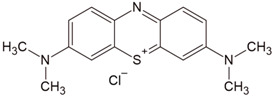	319.85	665
Methyl red sodium salt	[C_15_H_14_N_3_O_2_]^−^Na^+^	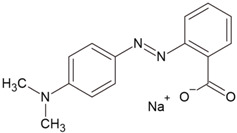	291.31	443

## Data Availability

Data is contained within the article.
